# Predicting Harms and Benefits in Translational Trials: Ethics, Evidence, and Uncertainty

**DOI:** 10.1371/journal.pmed.1001010

**Published:** 2011-03-08

**Authors:** Jonathan Kimmelman, Alex John London

**Affiliations:** 1 Biomedical Ethics Unit, Department of Social Studies of Medicine, McGill University, Montreal, Quebec, Canada; 2 Department of Philosophy, Carnegie Mellon University, Pittsburgh, Pennsylvania, United States of America

**Summary Points**

- Ethical judgments about risk, benefit, and patient eligibility in clinical trials hinge on predictions about harm, therapeutic response, and clinical promise.
- Predictions for novel interventions in preclinical stages of development suffer from two problems: insufficient attention to threats to validity in preclinical research and a reliance on an overly narrow base of evidence that includes only animal and clinical studies of the intervention in question (“evidential conservatism”).
- To improve ethical and scientific decision-making in early phase studies, decision-makers should explicitly attend to reporting quality and methodological features in preclinical experiments that address threats to internal, construct, and external validity.
- Decision-makers should also use evidence that sheds light on the reliability of causal claims embedded within a proposed trial. This evidence can be gathered from outcomes of previous trials involving agents targeting related biological pathways (“reference classes”).

## Introduction

First-in-human clinical trials represent a critical juncture in the translation of laboratory discoveries. However, because they involve the greatest degree of uncertainty at any point in the drug development process, their initiation is beset by a series of nettlesome ethical questions [1]: has clinical promise been sufficiently demonstrated in animals? Should trial access be restricted to patients with refractory disease? Should trials be viewed as therapeutic? Have researchers adequately minimized risks?

The resolution of such ethical questions inevitably turns on claims about future events like harms, therapeutic response, and clinical translation. Recurrent failures in clinical translation, like Eli Lilly's Alzheimer candidate semagacestat, highlight the severe limitations of current methods of prediction. In this case, patients in the active arm of the placebo-controlled trial had earlier onset of dementia and elevated rates of skin cancer [2].

Various authoritative accounts of human research ethics state that decision-making about risk and benefit should be careful, systematic, and non-arbitrary [3]–[5]. Yet, these sources provide little guidance about what kinds of evidence stakeholders should use to ensure their estimates of such events ground responsible ethical decisions. In this article, we suggest that investigators, oversight bodies, and sponsors often base their predictions on a flawed and inappropriately narrow preclinical evidence base.

## Prediction and Ethical Decision-Making

According to the core tenets of human research ethics, investigators, sponsors, and institutional review boards (IRBs) are obligated to ensure that risks to volunteers are minimized and balanced favorably with anticipated benefits to society and, if applicable, to the volunteers themselves [4],[6]. Accurate prediction plays a critical role in this process. When research teams underestimate the probability of favorable clinical or translational outcomes, they undermine health care systems by impeding clinical translation. When investigators overestimate the probability of favorable outcomes, they potentially expose individuals to unjustified burdens, which may be considerable for phase 1 studies involving unproven drugs. In both cases, misestimation threatens the integrity of the scientific enterprise, because it frustrates prudent allocation of research resources [7].

Naturally, there are limits to the reliability with which forecasts based on experimental evidence predict clinical outcomes. However, in late stages of clinical development, forecasts underwriting ethical and scientific decision-making have proven fairly reliable. Several analyses of cancer randomized controlled trials indicate that new interventions are just as likely to prove more effective than comparator ones as they were to prove inferior [8]–[10]. Similar findings have been reported for other indications [11]. In the aggregate at least, researchers and review committees neither overestimate nor underestimate the medical benefits of allocating some patients to new interventions and others to standard drugs.

Whether decision-makers utilize evidence as effectively when predicting outcomes in early phase research has not been systematically investigated. Nevertheless, there are grounds for concern such that a systematic investigation is overdue. Highly promising preclinical findings in cancer, stroke, HIV vaccines, and neurodegenerative diseases frequently fail clinical translation. In cancer, only 5% of products entering trials are eventually licensed [12],[13]. In one study, approximately 5% of high impact basic science reports were clinically translated within 10 years [14]. We suggest that these disappointments partly reflect two problems in the way evidence is used in predicting clinical outcomes.

## Preclinical Reporting and Validity

First, decision-makers may not be adequately responsive to problems in preclinical research practice [15]. Systematic reviews repeatedly demonstrate that many animal studies do not enable reliable causal inference and clinical generalization because they do not address important threats to internal, construct, and external validity. With respect to the first, one recent analysis of animal studies showed that only 12% used random allocation and 14% used blinded outcome assessment [16]. Construct validity concerns the relationship between clinical implementation of an intervention and implementations evaluated in preclinical studies. A recent review found that clinical studies of cardiac arrest interventions applied treatment significantly sooner after cardiac events than in preclinical studies [17]. In the case of Astra Zeneca's failed stroke drug NXY-059, use of normotensive rodents in preclinical development may have led to spurious predictions of clinical activity [18]. Preclinical studies do not always test the extent to which cause and effect relationships hold up under varied conditions (external validity). In a systematic review of neuroprotective agents in phase 2 and 3 trials, only two of ten agents were tested in both rodents and higher order species [19]. Finally, deficiencies in reporting and aggregation of preclinical evidence deprive decision-makers of crucial evidence. In one recent analysis, publication bias in preclinical stroke studies led to a 30% overestimation of treatment effect size [20]. Clearly, preclinical researchers should endeavor to follow reporting guidelines [21] such as the recently proposed Animals In Research: Reporting *In Vivo* Experiments Guidelines (ARRIVE; http://www.nc3rs.org.uk/page.asp?id=1357) [22], and clinical predictions following from animal studies should take into account deficiencies in design and reporting.

In the case of semegacestat, it has been over 5 years since the drug was first tested in human beings, and preclinical studies have yet to be published. However, narrative reviews by Eli Lilly scientists indicate trials were launched on the basis of molecular, rather than behavioral, endpoints [23]. Although the absence of publication makes difficult any assessment of animal study quality, the use of molecular endpoints raises questions about the construct validity of clinical generalizations drawn from preclinical experiments.

## Evidential Conservatism

A second concern about forecasting outcomes in translational trials relates to a tendency to base clinical inferences on a relatively narrow class of evidence: those preclinical studies that involve the particular agent. We call this “evidential conservatism.” Such evidential conservatism is reflected in various policies. For example, the American Society of Clinical Oncology states that “the decision to move an agent into phase I evaluation is based… central[ly on]… the observation of sufficient preclinical antitumor activity, such that a therapeutic effect in human cancer is anticipated” [24],[25]. International Council on Harmonization policy requires investigators to furnish ethics review committees with only a narrow type of preclinical evidence [26]. Similarly, some commentators argue that risk-benefit decisions in early phase trials should be driven by mechanistic evidence about an agent [27].

Evidential conservatism, however, fails to address the higher-order question of the reliability of forecasts made from such a narrow evidence base. This higher-order question is of special relevance for early phase research because agents that do not enjoy the support of promising preclinical results will not be plausible candidates for translation. Yet when agents are supported by equally promising preclinical results they may be differentiated by the maturity of the knowledge surrounding a nexus of variables concerning the relationship between test and target populations.

For instance, although neuroprotective stroke treatments have moved to translation on the basis of very encouraging preclinical studies, they have consistently failed randomized trials. Estimates of the risks and benefits of any particular neuroprotective compound that are based solely on preclinical evaluation of that compound will be less reliable than those that incorporate information about the relative success of neuroprotective compounds as a class. In part, this is because the success or failure of other interventions in this reference class provides evidence about the degree to which clinical development is guided by a reliable working knowledge of relevant disease processes.

Our claim that decision-makers need to use a broader base of evidence for evaluating early phase research is consistent with a recent call for incorporating whole research program outcomes into systematic reviews of particular agents [28].

## Assessing Relevant Evidence

How might researchers depart from evidential conservatisim in a way that is open to scrutiny and amenable to assessment, revision, and improvement? Decision-makers who make forecasts about agent activity in early phase research must identify reference classes that are relevant to the decision at hand. Delimiting the reference class of relevant evidence poses a challenge in that interventions possess limitless characteristics. A drug might be classed within neuroprotective compounds, stroke drugs, and drugs beginning with the letter “n.” Decision-makers thus confront the timeless problem of selecting those characteristics most salient for prediction.

There are no simple formulas here. In some cases, choice of reference classes will be straightforward (e.g., a new, small molecule HMG-CoA reductase inhibitor); in other cases, consensus may be elusive. Nevertheless, we suggest that the very act of attending to reference class identity would be a departure from evidential conservatism. As a starting place, decision-makers should identify reference classes that index the maturity of knowledge regarding central causal premises embedded within a protocol. In an era in which basic science heavily informs product development, drug developers themselves often class their agents according to explicit ambitions about causal pathways. Asserting that a drug targets a particular pathophysiologic process should prompt us to look at how other drugs that target the same process performed in clinical translation. We can then base our estimates of the maturity of knowledge about these causal premises on the success or failure of past attempts at redeeming these ambitions. Decision-makers should therefore adjust their confidence in clinical generalizations on the basis of outcomes with previous interventions that addressed the same pathological processes.

Semagacestat was screened and designed to target amyloid-β production, which is believed to be a key step in dementia onset. Eight other anti-amyloid drugs have either failed randomized trials or been abandoned due to toxicity (Table 1) [29],[30]. Although a variant of this approach may eventually succeed, promising preclinical evidence supporting semagecestat should have been tempered by the accumulation of data about outcomes in the same reference class.

**Table 1:** Outcomes in randomized trials of anti-amyloid drug candidates for Alzheimer's disease.

Drug	Phase	Outcome	Source
AN1792	2a	Unacceptable toxicity	[35]
Atorvastatin	3	No significance vs. placebo for two co-1° endpoints	[36]
AZD-103	2	No significance vs. placebo for two co-1° endpoints; toxicity	[37]
Bapineuzumab	2	No significance vs. placebo for 1° endpoint	[38]
Phenserine	3	No significance vs. placebo in efficacy analysis	[39]
Rosiglitiazone	3	No significance vs. placebo for two co-1° endpoints	[40]
Tarenflurbil	3	No significance vs. placebo for two co-1° endpoints	[41]
Tramiprostate	3	No significance vs. placebo for 1° endpoints	[42]

## Practical Implications

To illustrate how our suggestions interface with ethical decision-making, consider recent proposals to reinitiate trials of fetal-derived tissues for Parkinson's disease [31]. Previous trials involved treatment-refractory patients, but investigators are now proposing trials involving patients with recent onset. The rationale is that fetal-derived tissues can only protect dopaminergic neurons to the extent that the latter remain intact. However, the risk-benefit balance is contentious, because the trial will expose patients who can manage symptoms with standard treatments to the risks of neurosurgery, immunosuppression, and cell transplantation.

According to evidential conservatism, investigators and ethics bodies should evaluate the risk-benefit balance by consulting preclinical studies and the biological rationale for patient-subject selection. One commentator notes that, on the basis of preclinical studies showing the intervention is designed to address early disease processes, performing studies in patients with advanced disease would be unethical [27]. We think this way of using evidence in ethical evaluation is misguided.

Our proposal directs decision-makers to make risk-benefit decisions in light of two additional factors. First, to what degree do the preclinical studies incorporate design elements that support reliable inferences about clinical activity? This directs stakeholders to attend to those methodological features of the preclinical studies that support credible claims of internal, construct, and external validities in preclinical studies. As these preclinical studies are presently underway, researchers have an opportunity to overcome past limitations in addressing validity threats in Parkinson's disease models [32].

Second, our proposal directs stakeholders to consider evidence that sheds light on the maturity of the knowledge relating to key causal claims presupposed by therapeutic predictions. As investigators propose to intervene in degenerative processes, a claim of therapeutic action would need to be evaluated in light of outcomes in previous Parkinson's trials involving surgically delivered neuroprotective agents and/or transplanted tissues. No such strategies have produced positive randomized trials (Table 2). Accordingly, even with carefully collected preclinical evidence, decision-makers should approach new trials with modest therapeutic expectations.

**Table 2:** Outcomes in randomized trials of neuroregenerative and/or cell-transplantation strategies for Parkinson's disease.

Agent	Outcome	Source
CERE-120	No significance vs. sham on 1° endpoint	[43]
GDNF (intraventricular delivery)	No significance vs. sham on 1° outcome; toxicity	[44]
GDNF (initraputamenal delivery)	No significance vs. sham on 1° endpoint	[45]
Embryonic mesencephalic tissue	No significance vs. sham on 1° endpoint; toxicity	[46]
Embryonic mesencephalic tissues	No significance vs. sham on 1° endpoint; toxicity	[47]
Fetal porcine ventral mesencephalic tissue	No significance vs. sham	[48]
Retinal epithelial pigmented cells	No significance vs. sham	[49]

Thoughtful commentators have argued that, before initiating cell-based dopamine replacement, strategies should be “clinically competitive” with standard of care [33]. However, this may present an unworkable standard [34]. Previous unsuccessful attempts at translation betray profound uncertainty concerning risks and benefits for research volunteers. Given the preliminary nature of such interventions, the ethical justification for their administration in early phase trials should not hinge on the prospect of benefit for volunteers. It should rest instead on a compelling claim of knowledge value and on the reduction of avoidable risks. The latter entails pursuing trials in patients less likely to suffer opportunity costs from study participation, and maintaining a background of medical management that does not fall below standard of care. Rather than being told that the approach is comparable to standard of care, the consent process should emphasize that clinical benefit is unlikely.

## Conclusion

Systematic study of preclinical research has centered on stroke and practices focused on internal validity. Our proposal makes clear the need to broaden the scope of this research agenda to cover a wider range of preclinical research, and to expand its focus to include issues of construct and external validity. A key component of this process will involve creating databases for aggregating translational outcomes according to relevant reference classes.

Some may worry that such an analysis might produce less optimistic predictions, and hence stymie product development. However, we do not see how medicine is advanced by forging ahead on the basis of predictions of dubious reliability. Moreover, there are many productive ways in which stakeholders may respond to less optimistic projections. For instance, review of relevant information may prompt researchers to test certain hypotheses before moving ahead with human trials. Investigators might adjust the design of translational studies to align the risk profile with ethical judgments. Or, investigators might decide that moving forward with a protocol represents the best way to advance a particular scientific initiative, but that risks can only be justified by appealing to the value of the knowledge sought, rather than the product's therapeutic activity.

Stakeholders might already adjust their predictions in light of intuitions about validity or experiences with success or failure for similar agents. If so, they do so on the basis of private beliefs, and often without the data needed to make these adjustments systematically. Our approach provides a more publicly accessible basis for making and adjudicating risk-benefit predictions. We suggest that this would better cohere with a sage prescription offered by the National Commission: “there should first be a determination of the validity of the presuppositions of the research…. The method of ascertaining risks should be explicit… It should also be determined whether an investigator's estimates of the probability of harm or benefits are reasonable, as judged by known facts or other available studies ” [3].
